# Self-reported interoception, worries and protective behaviors during the COVID-19 pandemic: a longitudinal study

**DOI:** 10.1186/s41155-023-00267-x

**Published:** 2023-08-31

**Authors:** Luca Vig, Eszter Ferentzi, Ferenc Köteles

**Affiliations:** 1 Doctoral School of Psychology, ELTE Eötvös Loránd University, Budapest, Hungary; 2 Institute of Health Promotion and Sport Sciences, ELTE Eötvös Loránd University, Prielle Kornélia Utca 47-49, Budapest, 1117 Hungary; 3 Ádám György Psychophysiology Research Group, Budapest, Hungary; 4 Károli Gáspár University of the Reformed Church in Hungary, Budapest, Hungary

**Keywords:** Self-reported interoception, COVID-19 pandemic, Health protective behavior, Anxiety, Worry

## Abstract

**Background:**

Protective behaviors were essential for minimizing the spread of the virus during the coronavirus disease 2019 (COVID-19) pandemic. It is often assumed that awareness of bodily sensations (interoception) can improve decision-making and facilitate adaptive behavior.

**Objective:**

This paper investigates cross-sectional and longitudinal relationships between different aspects of self-reported interoception, trait anxiety, COVID-related worry, and health protective behaviors.

**Methods:**

The study was conducted on a community sample of 265 adults. The two data collection phases took place online, before (baseline) and during the second wave of the COVID-19 pandemic in Hungary.

**Results:**

Contrary to our expectations, neither cross-sectional nor longitudinal associations were found between protective behaviors and indicators of self-reported interoception. However, worry at baseline predicted protective behaviors during the second wave, even after controlling for socio-economical characteristics and protective behaviors at baseline.

**Conclusion:**

Our results highlight the adaptivity of health-related worry when behavioral steps to avoid threats are known and available. Also, higher level of perceived interoception did not appear to be health protective under these circumstances.

## Introduction

Interoception, i.e., the sense of the physiological condition of the body (Craig, 2002), is related to various factors of healthy functioning such as mental health and well-being (Farb et al., 2015; Ferentzi et al., 2019; Hanley et al., 2017; Khalsa et al., 2018; Luo et al., 2022), decision-making (Damasio, 1994; Dunn et al., 2010, 2012) and body regulation (Petzschner et al., 2021). To date, only a few studies have investigated interoception in the context of the coronavirus disease 2019 (COVID-19) pandemic (for details, see below) (Belhouk-Herrero et al., 2021; Elliott & Pfeifer, 2022; Vabba et al., 2022). In this longitudinal study, we explored how the self-reported (perceived) aspect of interoception, as measured with different questionnaires, is associated with COVID-19-related worries and protective behaviors.

There are several ways to pay attention to, perceive, interpret, and utilize one’s own bodily signals. For self-reported interoception, the conceptual difference between anxiety-related hypervigilance and mindful bodily focus (characterized by non-evaluative acceptance) has been highlighted (Mehling, 2016). Awareness of normal, non-emotive bodily processes is related to positive affect and well-being (Daubenmier, 2005; Ferentzi et al., 2019; Impett et al., 2006; Köteles, 2014; Luo et al., 2022; Moradi & Huang, 2008; Tihanyi et al., 2016), and largely independent from anxiety and symptom reporting tendency (Shields et al., 1989). Different aspects of mindful bodily attention, most importantly the tendency to experience psychical discomfort without worrying, the trust in one’s own body, and the ability to control and sustain attention consciously to bodily sensations show a strong negative association with anxiety-related constructs (Mehling, 2016). In the context of the COVID-19 pandemic, the ability to regulate distress by directing attention to physical sensations and trusting in one’s body were predictors of well-being (Vabba et al., 2022).

In contrast, hypervigilance to and constant monitoring of body sensations that possibly indicate pathology (i.e., symptoms) are associated with negative affectivity. For example, the awareness of stress-related bodily sensations was linked to anxiety about COVID-19 (Elliott & Pfeifer, 2022). An important representative of the negative aspect of interoception is somatosensory amplification, i.e., “the tendency to experience somatic sensation as intense, noxious, and disturbing” (Barsky et al., 1988, p. 510). As somatosensory amplification includes somatic hypervigilance and interpretation of sensations as signs of diseases, it is incorporated in the cognitive model of health anxiety (Abramowitz et al., 2007a, 2007b; Witthöft et al., 2020).

The sense of the bodily state lays the foundation for the homeostatic regulation (Paulus & Stein, 2010), which is manifested partly by physiological (e.g. sweating if the core temperature of the body is too high) and partly by behavioral (e.g. looking for a shady spot) changes. From an evolutionary point of view, awareness of the bodily state could facilitate adaptive behavior (Damasio & Carvalho, 2013). Concerning the early phases of COVID-19, protective behaviors had an essential role in preventing the spread of the virus in the lack of effective pharmaceutical treatments. Evidence shows that public health interventions (e.g. border restrictions, quarantine isolation, and social distancing) and behavioral changes of the public (including enhanced hygiene, face masks, and reduction of social contacts) slowed down the spread of the virus (Cowling et al., 2020). It is an interesting question whether self-reported interoception can improve decision-making and health protective behavior under such risky circumstances. For example, Bakal (1999) includes protective behavioral steps (decisions and lifestyle changes to achieve better health) in the definition of somatic awareness, a construct closely related to self-reported interoception. Also, it has been proposed that a higher level of body focus might facilitate early identification of symptoms of various diseases (Bakal et al., 2008); and non-judgmental body awareness is considered a health protective factor, related to self-care (Mehling et al., 2011). Regarding somatosensory amplification, from a novel perspective (Köteles & Witthöft, 2017) it primarily refers to the automatic emotional response to an internal or external threat to the integrity of the body, that gives rise to health and illness related cognition. If the risk of getting ill is comparatively high, monitoring the body for symptoms can be an adaptive strategy, as it enables the individual to deal with the threat in time (Abramowitz et al., 2007a, 2007b; Köteles & Simor, 2014; Lovas & Barsky, 2010). In addition, different theoretical models (e.g. Health Belief Model (Champion & Skinner, 2008), Protection Motivation Theory (Floyd et al., 2000; Rogers, 1975)) assume that perceived risk and worry about the possible consequences of the illness are important factors that motivate health protective behaviors. In line with this, fear of COVID-19, perceived risk and anxiety were consistently associated with public-health compliant behaviors in empirical studies (Dryhurst et al., 2020; Harper et al., 2021; Raude et al., 2020; Schneider et al., 2021; Urbán et al., 2021; Wise et al., 2020).

Some empirical results suggest that the subjective experience of bodily signals is indeed related to adaptive behavior and decision-making. The awareness of anxiety-related bodily signals was associated with higher risk-aversion in case of body-related decision-making (Salvato et al., 2019). Also, self-reported interoception had a significant modulating effect on decision-making under COVID-19-related stress (Belhouk-Herrero et al., 2021). A recent study (Arora et al., 2021) showed a positive association between sleep quality and the self-reported tendency of not distracting one’s attention from uncomfortable sensations and experiencing the body as trustworthy. A possible explanation of this finding is that individuals who do not ignore unpleasant sensations (e.g., hunger, pain, sleepiness) can take action in time which in turn leads to healthier sleep (Arora et al., 2021). On the other hand, Ginzburg and colleagues (2014) did not find an association between the self-reported sensibility to normal bodily processes and certain health-related behaviors (such as physical activity, vitamin, medication and alcohol consumption, and smoking).

Overall, the association between various aspects of self-reported interoception and protective behavioral changes in potentially threatening situations is a question of both theoretical and practical importance. In the present longitudinal study, we collected data before and during the second wave of the COVID-19 pandemic in Hungary, when vaccination was not yet available. It was hypothesized that aspects of self-reported interoception would show a positive association with protective behaviors (Hypothesis 1). Also, we intended to conceptually replicate the reported positive association between anxiety, COVID-19-related worries and health protective behaviors (Hypothesis 2).

## Materials and methods

### Participants

The online, Hungarian language survey was promoted in an online Psychology themed magazine. After indicating informed consent, participants could choose whether they would like to take part in the second phase of the research as well. (See the Process and context section below for details of data collection.)

Upon request, participants could receive feedback on their scores on the Multidimensional Assessment of Interoceptive Awareness (MAIA) (Mehling et al., 2012) after the data collection period. 392 individuals started to fill out the survey, 127 were excluded because they completed less than 30% of items of the entire test battery. The final sample consisted of 265 individuals (222 female, mean age: 38.2 ± 11.6). Out of them, 151 filled out the survey via email in the second phase. Of all participants, 0.8% had elementary school level education, 20.8% finished high-school, 78.5% had university diploma. Part of the hereby analyzed dataset (cross-sectional data on MAIA and the Body Awareness Questionnaire (BAQ)) was used in a previous study (Vig et al., 2022). The study was approved by the Ethical Board of the University (Approval Nr. 2020/289).

### Instruments

The Body Awareness Questionnaire (BAQ) (Shields et al., 1989) measures the perceived sensibility to normal (i.e. non-pathological), non-emotive bodily processes. It focuses on bodily rhythms and cycles, the ability to detect small bodily changes, and the ability to anticipate bodily reactions (e.g., “I can accurately predict what time of day lack of sleep will catch up with me”). The Hungarian version (Köteles, 2014) comprises 17 statements, that are rated on a 7-point Likert scale (1 = not at all true about me, 7 = very true about me). The Cronbach’s alpha coefficient of the questionnaire in this study was 0.82.

The Multidimensional Assessment of Interoceptive Awareness (MAIA) (Ferentzi et al., 2020; Mehling et al., 2012) measures different, adaptive aspects of self-reported interoception on 8 scales (Noticing, Not Distracting, Not Worrying, Attention Regulation, Emotional Awareness, Self-Regulation, Body Listening, and Trusting). Based on their assumed relevance in behavior regulation, five scales were applied. The Noticing scale measures the awareness of neutral, uncomfortable, and comfortable bodily sensations with 4 items. The Not Distracting scale refers to the tendency of not using distraction as a method to cope with uncomfortable sensations and pain with 3 items. The Not Worrying scale quantifies the tendency of not reacting with emotional distress to discomfort and pain with 3 items. The Body Listening scale assesses the tendency of actively listening to the body for insight with 3 items. Finally, the Trusting scale refers to the extent to which one experiences their body trustworthy and safe with 3 items. Respondents have to rate their level of agreement with each statement on a 5-point Likert scale (1 = Never, 5 = Always). (Example item:” I feel my body is a safe place.”) Internal consistency values of the included scales in this study ranged from 0.65 to 0.87 (Noticing: 0.73; Not Distracting: 0.65; Not Worrying: 0.76; Body Listening: 0.80; Trusting: 0.87).

The Somatosensory Amplification Scale (SSAS) (Barsky et al., 1990; Köteles et al., 2009) measures the proneness to experience somatic sensation as intense, noxious, and disturbing. We consider the construct a measure of self-reported interoception. Agreement with 10 statements is rated on a 5-point Likert scale (1 = not at all; 5 = extremely). Items cover uncomfortable, but usually not directly illness-related sensations (e.g., „I hate to be too hot or too cold.”). Cronbach’s alpha coefficient of the scale in this study was 0.66.

The Spielberger State-Trait Anxiety Inventory (STAI) (Sipos et al., 1994; Spielberger et al., 1970) differentiates between state and trait aspects of anxiety. While the former shows how the respondent feels at the moment, the latter refers to the level of anxiety as a temporally stable characteristic. In the present study the trait anxiety scale of the questionnaire (STAI-T) was used, which consists of 20 statements about the general state of mind. The level of agreement with each statement is scored on a 4-point Likert-scale (1 = not at all; 4 = fully). (Example item:” I am inclined to take things hard.”). Cronbach’s alpha coefficient of the scale in this study was 0.92.

COVID-related worry was measured with 3 questions in both data collection phases, i.e., “How worried are you about the coronavirus right now?”, “How likely do you think it is that you will be infected with the coronavirus?”, and “How afraid are you that if you get infected, you could develop a serious illness due to the coronavirus?” Items were rated on a 100-point visual analogue scale. Cronbach’s alpha of these questions was 0.68 at the first data collection phase and 0.67 at the second.

COVID-19-related protective behaviors were measured in both data collection phases with yes-or-no questions, based on the list of Jungmann and Witthöft (2020). It includes 13 items: internet research, visits to doctors, increased shopping for hygiene products/food, purchase of face mask, increased washing of hands, increased use of disinfection, wearing a face mask, taking food supplements, avoiding crowds > 100 people, avoiding major events > 1000 people, avoiding travel within/outside Hungary. The total score of the answers was used in the study. One item was removed from the original version (about stealing hygiene products/face masks from a hospital or other institution). Increased shopping for food was excluded from the final analysis as it lacks health protective value.

### Process and context

The data was collected in two phases online. The questionnaires were filled out only in the first data collection period. COVID-19-related worries and behaviors were assessed in both data collection phases.

The World Health Organization (WHO) declared an outbreak of COVID-19 at the end of January 2019, and a pandemic on the 11^th^ of March 2020. Hungary had the first registered case on the 4^th^ of March 2020. The government declared a state of emergency on the 11^th^ of March 2020 which lasted until the 18^th^ of June 2020. Overall, the time period from the 4^th^ of March to the 17^th^ of July 2020 was considered the first wave of the pandemic in Hungary (Uzzoli et al., 2021).

The first, baseline data collection (Time 0) was conducted between the 17^th^ of July and the 27^th^ of August 2020, when a longer interim period between the first and the second wave took place (Uzzoli et al., 2021). During this period, the previous strict restrictions were released, and people’s everyday activity returned to almost normal.

The second data collection phase (Time 1) took place between the 23^rd^ of November and the 2^nd^ of January 2021. In Hungary, the second wave of COVID-19 is dated from October–November 2020 to the 16^th^ of February 2021 (Kovalcsik et al., 2021). During the second wave, strict restrictions were imposed again. Some of the most important measures were nighttime curfew, prohibition of all gatherings, disallowing restaurants, and hotels (with some exceptions) from having guests, digital education from the 9^th^ grade, in universities and colleges, closing of museums, swimming pools, libraries, cinemas, zoos, and cancellation of cultural events. The most important behavioral recommendations were communicated on billboards: wash your hands, keep a distance of 1.5 m, and wear a mask.

### Statistical analysis

Statistical analysis was conducted using the Jasp v0.16.3. software (JASP Team, 2022). Differences between worry and protective behavior at Time 0 and Time 1 were tested with Wilcoxon signed-rank test, effect size was estimated with rank-biserial correlation; associations between these variables were estimated with Spearman correlation. Also, cross-sectional and longitudinal associations between worry/behavior and questionnaire scores were estimated with Spearman correlation. In order to avoid the accumulation of Type 1 error, accepted level of significance was set to *p* < 0.001 for the cross-sectional analysis (72 independent tests), and *p* < 0.003 for the longitudinal analysis (16 independent tests). Predictive power of worry at baseline and the assessed self-report variables was examined with separate multiple linear regression analyses with behavior at Time 1 as criterion variable. All equations were controlled for sex, age, educational qualification, and behavior at baseline.

## Results

Descriptive statistics are presented in Table 1. It is important to emphasize that all variables are characterized by a high level of variance; thus, results of the analysis are not limited by the homogeneity of the sample.

**Table 1.** Descriptive statistics of the assessed variables

	N	M	SD	min	max
SSAS	225	31.34	5.71	17	47
BAQ	226	85.1	12.16	48	119
STAI-T	230	45.04	9.49	23	69
MAIA Noticing	233	3.75	0.81	1	5
MAIA Not Worrying	233	2.93	0.95	1	4.67
MAIA Not Distracting	233	3.04	0.85	1	5
MAIA Body Listening	233	3.20	0.96	1	5
MAIA Trusting	232	3.83	0.88	1	5
Worry at t0	265	36.36	20.42	0	93
Worry at t1	151	46.76	20.59	0	94
Behaviors at t0	265	4.92	2.58	0	11
Behaviors at t1	151	6.39	2.68	0	11

*Note*: *SSAS* Somatosensory Amplification Scale, *BAQ* Body Awareness Questionnaire, *STAI-T* Trait Anxiety Inventory, *MAIA* Multidimensional Assessment of Interoceptive Awareness

### Worry and behaviors

**Fig. 1:**
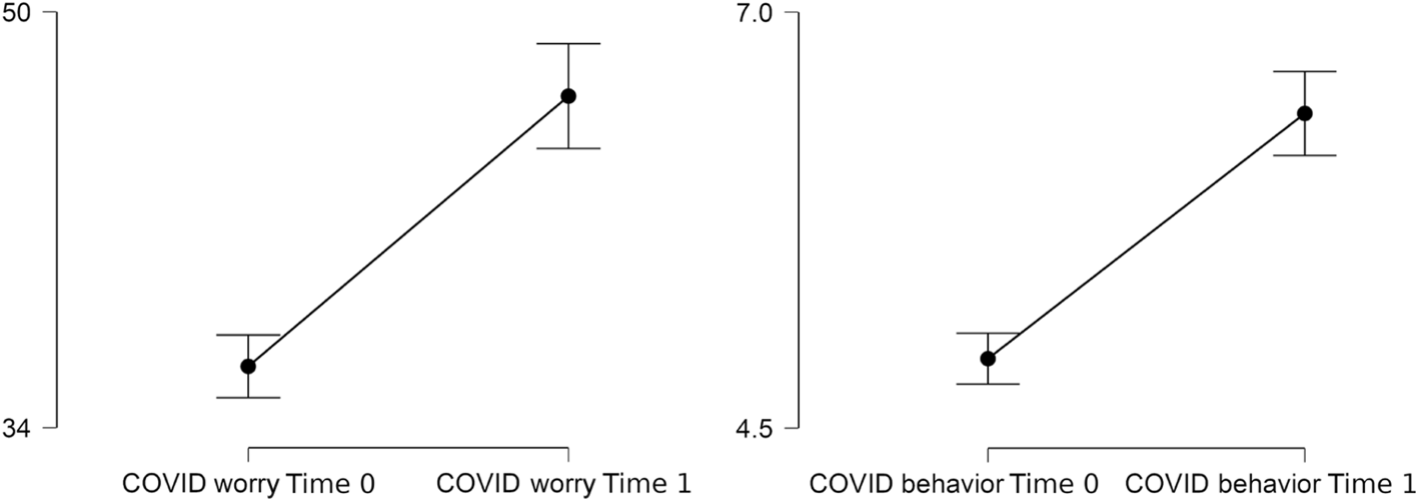
Changes in worry and protective behaviors from Time 0 to Time 1. Error bars indicate 95% CIs

Both worry (*W* = 2432.5, *p* < 0.001, *r* = -0.553) and protective behaviors (*W* = 1365.5, *p* < 0.001, *r* = 0.648) showed a significant increase with large effect size from Time 0 to Time 1 (Fig. 1).

Worry and behaviors showed a strong positive cross-sectional association at Time 0 (*r*_*s*_ = 0.552, *p* < 0.001). Concerning longitudinal associations, worry at Time 0 was a strong predictor of worry at Time 1 (*r*_*s*_ = 0.589, *p* < 0.001), and was moderately related to behaviors at Time 1 (*r*_*s*_ = 0.418, *p* < 0.001). The latter association remained significant after controlling for age, sex, educational qualification, and behaviors at Time 0 (Table 2); the regression equation explained 41.4% of the total variance (*F* (5,145) = 20.509, *p* < 0.001). The association between behaviors at Time 0 and Time 1 was strong (*r*_*s*_ = 0.615, *p* < 0.001).

**Table 2:** Output of multiple linear regression analysis with protective behaviors at Time 1 as criterion variable

	B ± SE	95% CIs	Standardized β	*p*
sex	0.191 ± 0.475	-0.747—1.129	0.026	0.688
age	2.452e-4 ± 0.017	-0.033—0.033	0.001	0.988
educational qualification	0.381 ± 0.468	-0.544—1.307	0.056	0.417
Worry at Time 0	0.021 ± 0.010	9.739e-4—0.040	0.148	0.040
Behaviors at Time 0	0.607 ± 0.079	0.451—0.763	0.550	< .001

### Indicators of anxiety and interoception

Cross-sectionally, worry showed a weak positive association with STAI-T and a weak negative association with MAIA Not Worrying. Protective behaviors were not significantly related to any questionnaire scores (for details, see Table 3).

**Table 3:** Cross-sectional associations (Spearman-correlations) between indicators of interoception and trait anxiety, worry, and protective behaviors at Time 0

Variables at baseline	Worry	Behaviors	1	2	3	4	5	6	7
1. SSAS	0.115	0.174							
2. BAQ	-0.120	-0.045	0.114						
3. STAI-T	0.249*	0.108	0.319*	-0.236*					
MAIA									
4. Noticing	-0.068	-0.014	0.032	0.415*	-0.214				
5. Not-Worrying	-0.278*	-0.179	-0.384*	0.087	-0.396*	0.240*			
6. Not Distracting	0.041	0.043	0.071	0.131	-0.157	0.188	-0.018		
7. Body Listening	0.004	0.164	0.116	0.373*	-0.224*	0.521*	0.081	.248*	
8. Trusting	-0.160	-0.053	-0.193	0.232*	-0.553*	0.353*	0.294*	0.187	0.451*

*Note*: *SSAS* Somatosensory Amplification Scale, *BAQ* Body Awareness Questionnaire, *STAI-T* The trait anxiety scale of the Spielberger State-Trait Anxiety Inventory, *MAIA* Multidimensional Assessment of Interoceptive Awareness

^*^p < .001

Longitudinally, worry at Time 1 was predicted by MAIA Not Worrying (moderate negative association). Protective behaviors at Time 1 were not associated with any indicators of interoception (for details, see Table 4).

**Table 4:** Longitudinal associations (Spearman-correlations) between indicators of interoception and trait anxiety at Time 0, worry at Time 1, and protective behaviors at Time 1

	SSAS	BAQ	STAI-T	MAIA				
				Noticing	Not Worrying	Not Distracting	Body Listening	Trusting
Worry at Time 1	0.111	0.023	0.149	-0.094	-0.338*	0.022	-0.028	-0.174
Behaviors at Time 1	0.068	-0.046	0.103	-0.048	-0.168	-0.036	0.067	-0.117

*Note*: *SSAS* Somatosensory Amplification Scale, *BAQ* Body Awareness Questionnaire, *STAI-T* The trait anxiety scale of the Spielberger State-Trait Anxiety Inventory, *MAIA* Multidimensional Assessment of Interoceptive Awareness

^*^p < .003

However, after controlling for socio-demographic characteristics and behaviors at Time 0, none of the assessed trait variables predicted behaviors at Time 1 (Table 5; all equations were significant (*p* < 0.001) and explained 38.2 to 39.4% of the total variance).

**Table 5:** Output of eight separate multiple linear regression analyses with protective behaviors at Time 1 as criterion variable and various indicators of interoception or trait anxiety at Time 0 as independent variable. Each equation was controlled for sex, age, educational qualification, and protective behaviors at Time 0

independent variable	B ± SE	95% CIs	Standardized β	p
SSAS	-0.015 ± 0.033	-0.080—0.051	-0.031	0.657
BAQ	-0.018 ± 0.017	-0.050—0.015	-0.076	0.287
STAI-T	0.014 ± 0.019	-0.024—0.051	0.050	0.466
MAIA				
Noticing	-0.087 ± 0.219	-0.520—0.347	-0.027	0.692
Not Worrying	-0.006 ± 0.199	-0.399—0.388	-0.002	0.977
Not Distracting	-0.082 ± 0.220	-0.516—0.352	-0.025	0.708
Body Listening	-0.139 ± 0.208	-0.551—0.272	-0.047	0.504
Trusting	-0.164 ± 0.220	-0.598—0.271	-0.050	0.457

*Note*: *SSAS* Somatosensory Amplification Scale, *BAQ* Body Awareness Questionnaire, *STAI-T* The trait anxiety scale of the Spielberger State-Trait Anxiety Inventory, *MAIA* Multidimensional Assessment of Interoceptive Awareness

## Discussion

In a longitudinal study in a community sample, the associations between self-reported interoception, trait anxiety, COVID-related worry and health protective behaviors were investigated before and during the second wave of the COVID-19 pandemic in Hungary. We expected that 1) self-reported aspects of interoception (BAQ, SSAS, MAIA Noticing, MAIA Not Worrying, MAIA Not Distracting, MAIA Body Listening, and MAIA Trusting), and 2) anxiety and worries would be cross-sectionally and longitudinally associated with protective behaviors. Contrary to our first hypothesis, however, no significant cross-sectional associations were found between protective behaviors and indicators of interoception. In addition, none of the trait-like variables (i.e., indicators of interoception and trait anxiety) predicted change in protective behaviors at Time 1. Overall, this pattern suggests that self-reported interoception did not directly impact protective behavior during the pandemic. In accordance with the second hypothesis, moderate to strong positive cross-sectional associations between COVID-related worry and protective behaviors were found. Also, worry at baseline predicted change in protective behaviors at Time 1 even after controlling for socio-economical characteristics and protective behaviors at Time 0. Furthermore, worry showed a weak positive cross-sectional association with STAI and a weak negative cross-sectional association with MAIA Not Worrying. Longitudinally, lower levels of COVID-related worries at Time 1 were predicted by MAIA Not Worrying. This scale of MAIA measures the proneness to handle uncomfortable sensations and pain without emotional distress and has a strong negative association with anxiety-related constructs (Mehling, 2016) and negative affectivity (Vig et al., 2022). These findings are in accordance with the idea that worry can be considered the cognitive aspect of anxiety (Mathews, 1990). It is also possible that the association between COVID-related worry and MAIA Not Worrying is the consequence of an underlying third variable, namely general worrying tendency.

Regarding the lack of association between adaptive aspects of self-reported interoception (assessed with five scales of the MAIA and the BAQ) and protective behaviors, a possible explanation is provided by the predictive-coding framework (Farb et al., 2015), i.e., the mindfulness-related aspect of interoception promotes perceptual inference instead of active inference. In other words, when noticing an uncomfortable bodily state, mindful attention style leads to modification of the expected state and acceptance of the sensed state rather than motivating behavioral change in order to reach the expected state. However, somatosensory amplification, which is related to risk perception (Köteles & Witthöft, 2017), showed no association with health protective behaviors either. Another possibility is that the link between interoception and health protective behaviors develops after the appearance of symptoms of a disease but not in a completely preventive (predictive) way. In addition, when interpreting the longitudinal results, we need to take into consideration that although self-reported interoception is often considered a trait-like characteristic (Ferentzi et al., 2018; Khalsa et al., 2018), i.e., a comparatively high temporal stability is assumed, it was recently found that it changed significantly during the COVID-19 pandemic (Vabba et al., 2022). Hence, during the second wave, the perception of the bodily state and even the level of state anxiety could be significantly different from that we measured at the baseline. It is also important to emphasize that the high temporal stability of protective behaviors considerably reduced the variance that could be explained by other variables.

Despite of the temporal stability of both constructs, positive associations between COVID-related worries and health protective behaviors were found cross-sectionally and longitudinally. The cross-sectional association refers to an interim period after the first COVID-19 wave, reflecting a situation, where the previous strict regulations were released, and everyday activities could go back to quasi-normal. The longitudinal association shows that COVID-related worries during this interim period predicted how much one engaged in protective behaviors during the second wave. This points out the adaptiveness of health-related worry in situations in which effective behavioral steps to avoid the threat are known and available. Harper (2021) argues that anxiety in the context of the COVID-19 pandemic is a normative and protective response to a dangerous situation that we cannot fight or escape, and our findings support this view. Risk perception and anxiety of COVID-19 were found to be associated with more protective behaviors in other studies too (Dryhurst et al., 2020; Harper et al., 2021; Raude et al., 2020; Schneider et al., 2021; Urbán et al., 2021; Wise et al., 2020). Recent studies also showed that people tend to optimistically underestimate the risk of infection compared to the “average person” (Kuper-Smith et al., 2021; Wise et al., 2020); this bias could have very harmful, even lethal consequences in this context. On the other hand, we should not ignore mental health problems, including anxiety-related issues and health anxiety induced by the COVID-19 pandemic (Al-Rahimi et al., 2021; Jungmann & Witthöft, 2020; Kibbey et al., 2021; Li et al., 2020; Qiu et al., 2020; Wang et al., 2020), and the adverse effects of the preventive measures such as isolation and quarantine (Witthöft et al., 2022).

It can be concluded that during the pandemic the quantity of health protective behaviors was associated with worries about the risks of COVID-19 and not with the self-reported aspects of interoception. This questions the assumed protective and preventive role of interoception (bodily focus or somatic awareness) (Bakal, 1999) when the health risks are high. In the lack of symptoms that indicate threat, even somatosensory amplification tendency did not facilitate adaptive behavior. The findings suggest the practical importance of having access to clear and balanced information about the health risks during the pandemic Hopefully, the results of this study could contribute to inform effective public health strategies and interventions.

### Limitations

First, our study relies on self-report, which can be affected by response bias, specifically when it comes to preventive behaviors. Second, the sum of the number of preventive behaviors was used and this score does not differentiate adaptive actions from extreme, maladaptive safety-seeking behavior. Notably, extreme levels of engaging in preventive behaviors can be harmful to the individual and the community (e.g., stockpiling hygiene products; see Asmundson &Taylor, 2020). Third, data from a comparatively small, non-representative community sample was analyzed, which restricts the generalizability of the results. The characteristics of the sample (83.7% female, 78.5% highly educated, with a probable interest in psychological topics) could lead to biased results. Fourth, we used three questions only to measure COVID-related worries; since then standardized measures of COVID-related distress were not yet available at time of the data collection (Ahorsu et al., 2022). Finally, the internal consistency of the Somatosensory Amplification Scale was quite low. This is, however, a frequent finding in the literature which might reflect the heterogeneity of the construct (Köteles & Witthöft, 2017).

## Conclusion

Worry about the harmful effects of COVID-19 predicted protective behaviors during the pandemic. Self-reported interoception, however, was unrelated to protective behaviors.

## Data Availability

The data that support the findings of this study are openly available on OSF (https://osf.io/9zmw2/).

## Abbreviations

BAQ: Body Awareness Questionnaire
COVID-19: Coronavirus disease 2019
MAIA: Multidimensional Assessment of Interoceptive Awareness
SSAS: Somatosensory Amplification Scale
STAI-T: The trait anxiety scale of the Spielberger State-Trait Anxiety Inventory
Time 0: Time of baseline data collection
Time 1: Time of second data collection
WHO: World Health Organization
